# Analysis of Sports Supplement Consumption of Elite Referees of the Spanish Professional Fotball League

**DOI:** 10.3390/nu16152486

**Published:** 2024-07-31

**Authors:** Antonio Jesús Sánchez-Oliver, Víctor Moreno-Pérez, Pablo Terrón-Manrique, Vicente Fernández-Ruiz, Iñaki Quintana-Milla, Javier Sánchez-Sánchez, Guillermo Rodríguez, Juan José Ramos-Álvarez, Raúl Domínguez, Álvaro López-Samanes

**Affiliations:** 1 Departamento de Motricidad Humana y Rendimiento Deportivo, Facultad de Ciencias de la Educación, Universidad de Sevilla, 41004 Sevilla, Spain; sanchezoliver@us.es (A.J.S.-O.); rdherrera@us.es (R.D.); 2 Sports Research Centre, Miguel Hernandez University of Elche, 03202 Alicante, Spain; vmoreno@umh.es; 3 Faculty of Health Sciences, Universidad Francisco de Vitoria, 28223 Madrid, Spain; p.terron.prof@ufv.es (P.T.-M.); vicente.fernandez@ufv.es (V.F.-R.); 4 Facultad de Ciencias de la Salud, Universidad Alfonso X el Sabio (UAX), Avenida de la Universidad, 1, 28691 Madrid, Spain; iquintanag@uax.es; 5 School of Sport Sciences, Universidad Europea de Madrid, 28670 Madrid, Spain; javier.sanchez2@universidadeuropea.es; 6 Comité Técnico de Árbitros (CTA) de la Real Federación Española de Fútbol (RFEF), 28232 Las Rozas, Spain; guillermo.rodriguez@rfef.es; 7 Faculty of Medicine, School of Sport Medicine, Madrid Complutense University, 28040 Madrid, Spain; jjramosa@med.ucm.es; 8 GICAF Research Group, Education, Research Methods and Evaluation Department, Faculty of Human and Social Sciences, Universidad Pontificia Comillas, 28049 Madrid, Spain

**Keywords:** sports nutrition, soccer, dietary supplements, referees, questionnaires

## Abstract

Background: Sports supplements (SSs) are widely used among team sport athletes; however, evidence supporting the use of SSs among football referees at the elite level is scarce. The aim of the present study was to analyze the consumption of SSs among referees with respect to their level of competition and referee type (main referees (MRs) vs. assistant referees (ARs)). Methods: A total of 106 football referees participated in this study, with 46.2% from the First Spanish Division and 53.8% from the Second Spanish Division, representing 84.13% of the total number of referees. Each participant completed a validated questionnaire about SS consumption, with the SSs classified according to the Australian Institute of Sport (AIS) ABCD system: Group A has strong evidence for enhancing athlete health and performance, Group B shows potential benefits but needs more evidence, Group C has inconclusive evidence against use, and Group D includes prohibited substances. Results: A total of 84.0% of the MRs and ARs reported the consumption of at least one SS. Differences were found only in the consumption of medical supplements between division (*p* = 0.016) and type of referee (*p* = 0.041), though no significant differences were found among the remaining AIS SS categories (*p* > 0.05). Sport performance (49.6%), Internet (41.0%), and dietitian–nutritionists (31.7%) were the primary reason for SS consumption, purchase location, and source of information, respectively. The most frequently consumed SS were whey protein (45.3%), followed by creatine (33.0%), sport bars and sports drinks (28.3%), and caffeine (19.8%). Conclusions: MRs and ARs reported a high prevalence of dietary supplement (SS) consumption, with significant differences between division and referee type observed only in medical supplement consumption.

## 1. Introduction

Football (soccer) stands out as one of the preeminent team sports globally, with millions of fans and participants worldwide [1]. As evidenced by Fédération Internationale de Football Association (FIFA), over 200 member associations are affiliated in this team sport, and the active involvement of approximately 250 million players worldwide underscores its widespread popularity [2]. There are a wide variety of people that are involved in football both directly and indirectly, such as players, technical staff, and referees [3]. Football referees are fundamental during football competitions since they oversee making decisions to apply the rules of the game. Normally, a main referee, two assistant referees, and a fourth official referee are required for a football match [2,3].

The main referees cover distances of between 10 and 12 km during official matches, with approximately 20% of the total distance characterized by high-speed running (i.e., speeds ranging between 18.0 and 24.9 km/h) [4], while assistant referees cover approximately half of this distance during competitive matches (i.e., ≈6 km per match) [5]. Due to the high-intensity demands on main referees and assistant referees during football matches, maintaining optimal physical fitness [4,6] and adhering to proper nutrition practices are paramount [2]. In the nutrition field, sports supplements (SSs) are commonly used in team sport disciplines (e.g., rugby, handball, and football), with use ranging between 59.9 and 87.2% [7,8,9,10,11]. However, the scientific knowledge of the SS consumption of referees and football referees is limited. Dietary supplements are defined as foods, food components, nutrients, or nonfood compounds that are intentionally ingested in addition to the regularly consumed diet, with the explicit aim of achieving a specific health or performance benefit [12] that could optimize the performance and maintain a proper health status of main and assistant referees, as previously reported in football players [10,13]. Some scientific institutions, such as the Australian Institute of Sport (AIS) and International Olympic Committee (IOC), have created different classifications for establishing the efficacy of dietary supplements. Specifically, the AIS presented the ABCD system, in which dietary supplements are classified according to the level of scientific evidence: Group A (i.e., subdivided into medical supplements, ergogenic aids, and sports foods) has a high level of scientific evidence for enhancing health performance in athletes; Group B includes dietary supplements with possible positive effects, but more evidence is still needed; Group C includes dietary supplements with inconclusive evidence against their use; and Group D includes prohibited substances [14].

To the best our knowledge, only a few studies in the field of referee nutrition have focused on understanding macronutrient and micronutrient consumption using a 3-day food diary and 24 h recall [15,16]. Additionally, only one study explored dietary supplement knowledge among a small group of referees in the context of football [17]. Thus, the aim of this study was to establish the SS consumption of elite Spanish football referees and the possible differences found in SS consumption between categories (i.e., First vs. Second Spanish Division Football League) or referee type (i.e., main referees vs. assistant referees).

## 2. Materials and Methods

### 2.1. Participants

A total of 106 professional male football referees in Spain voluntarily participated in this study. A total of 46.2% (*n* = 49) were referees from the First Spanish Division, and 53.8% (*n* = 57) were from the Second Spanish Division. In terms of referee type, 64.2% were assistant referees (AR) (*n* = 68), and 35.8% were main referees (MR) (*n* = 38). It should be noted that the total sample represented 84.13% of the total number of referees in both leagues. The reasons for nonparticipation were as follows: illness (*n* = 2), absences from the Royal Spanish Football Federation, absence at the time of data collection (*n* = 14), early departure from the association (*n* = 1), and refusal to participate in the study (*n* = 3).

### 2.2. Instruments

The questionnaire employed consisted of three main sections dedicated to anthropometric and personal information (in the first section); sports modality practices, which included questions concerning aspects such as category and type of practices, experience of practitioners, weekly training time, and SS consumption (in the second section), and a segment focused on factors related to the consumption of SSs (in the third section). Specifically, the third section comprised questions about SS consumption, including motivations, received advice, number of days they are consumed, timing of consumption, and purchasing locations, in addition to two questions regarding the use of prohibited substances. A definition of sports supplements and an updated list of these supplements were included. The response options allowed participants to specify the timing and purpose of supplement consumption [18]. Additional information regarding the instrument and its user manual can be consulted in the user guide developed by Sanchez-Oliver et al. (2024) [19].

The questionnaire was previously validated for its content, application, structure, and presentation [18]. Its quality was assessed in a meta-analysis, where it was 1 of the 57 questionnaires (out of a total of 164) considered suitable for obtaining precise information about supplement use [20]. To categorize the quality of the questionnaires, Knapik et al. (2016) employed an eight-point scale that considered factors such as sampling, sample size, measurement tools, bias, response rate, statistical presentation, and description of the participant sample [20]. Moreover, this questionnaire has been utilized in numerous studies that have investigated SS consumption among athletes [7,8,21,22].

### 2.3. Procedures

The administration of the questionnaire was conducted individually and face-to-face with all the referees and assistant referees that participated during the study. Data collection took place during the 2021–2022 season at one of the professional referees’ training camps organized by the Royal Spanish Football Federation. Throughout the data collection process, an expert in the field was present to clarify any uncertainties and guide the procedure, ensuring the accurate submission of responses. Upon arrival, participants were informed about this study’s objectives and characteristics, and consent was obtained from all participants. The protocol adhered to the Declaration of Helsinki for human research and received approval from the participants, who gave their informed written consent to participate, and this study was approved by the Ethics Committee of the Alfonso X El Sabio University (number 01/2017) in accordance with the Declaration of Helsinki.

### 2.4. Statistical Analysis

All qualitative variables are presented as percentages. To analyze potential differences based on referee type (i.e., referees vs. assistant referees) and category (i.e., First vs. Second Spanish Division) in the qualitative variables, a chi-squared test (χ²) was conducted. Additionally, for each association, the odds ratio (OR) with 95% confidence interval (CI) was calculated, considering IC ≥ 1.00 as a relative risk factor and a value lower than 1.00 in the upper value of the IC as a relative protective factor [23]. For the analysis of potential differences in the dietary supplements (SSs) consumed by the sample, only those SSs with a prevalence of consumption exceeding 10% of the sample were considered. Quantitative variables are presented as mean (M) ± standard deviation (SD). The Kolmogorov–Smirnov and Levene tests were utilized to assess the normality of distribution and homoscedasticity, respectively. Subsequently, a two-way ANOVA was conducted for the factors of referee type (i.e., referees or assistant referee) and category (i.e., First or Second Spanish Division) including the interaction between type and category. If statistical differences were observed, a post hoc Bonferroni test was applied. Statistical significance was established at *p* < 0.05. All statistical analyses were performed using Statistical Package for Social Sciences (SPSS) software (version 23.0, SPSS Inc., Chicago, IL, USA).

## 3. Results

The descriptive data for the sample are presented in Table 1. Concerning age, significant differences were noted based on referee type (F = 4.997; *p* < 0.001), competition level (F = 11.235; *p* = 0.028), and the interaction between referee type and competition (F = 4.344; *p* = 0.040). Specifically, the ARs in the Second Spanish Division were younger compared to their counterparts in the First Spanish Division (*p* < 0.001). Among these ARs, there were significant age differences compared with the MRs in the Second Spanish Division (*p* < 0.001), but there were no statistically significant differences between referees across different categories or between types within *the* First Spanish Division (*p* > 0.05). Regarding anthropometric data, MRs were heavier across both competition levels (F = 8.808; *p* < 0.05). Regarding height, significant differences were found based on referee type (F = 9.448; *p* = 0.003) and the interaction between referee type and competition category (F = 4.203; *p* = 0.043). MRs were taller than the ARs in the Second Spanish Division (*p* < 0.003). However, no significant differences in height were observed within the First Spanish Division (*p* = 0.491). The ARs from the Second Spanish Division were shorter in comparison with those from the First Spanish Division (*p* = 0.022). No differences were detected in the frequency of weekly training sessions based on referee type, competition level, or the interaction between referee type and competition (Table 1).

Regarding SS consumption in the season in which the data were recorded, 84.0% of the sample reported using SSs, with no significant differences between MRs and ARs (*p* = 0.595) or between the First and Second Spanish Divisions (*p* = 0.116). Although no significant differences were detected in the prevalence of consumption, MRs reported a higher perceived effect of SS consumption on a Likert scale compared to ARs (3.99 ± 0.12 vs. 3.68 ± 0.96; *p* = 0.046), with this difference reaching statistical significance in the Second Spanish Division (*p* = 0.012, but not in the First Spanish Division (*p* = 0.790).

Concerning the reasons for SS intake, 49.6% of respondents cited enhancing sports performance, and 38.2% cited improving health status, followed by 9.2% for esthetic reasons. No significant differences were found regarding these motivations based on either referee type (*p* = 0.164) or competition level (*p* = 0.686). Similarly, no significant differences were found in purchase location based on referee type (*p* = 0.490) or division category (*p* = 0.867), with 41.0% buying online, 19.7% at pharmacies, and 17.2% from specialized stores. The most frequent advisors for sports supplement intake were dietitians/nutritionists (31.7%), followed by physical trainers (25.4%) and other referees (15.9%), with no significant differences observed in advisor influence by referee type (*p* = 0.204) or category (*p* = 0.867).

Regarding consumption days, 41.1% of the sample consumed SSs on both training and competition days, 30.0% only on training days, 16.7% every day (rest days included), and 12.2% only on competition days. No significant differences were found based on referee type (*p* = 0.426) or category (*p* = 0.855). Concerning timing, 42.2% ingested SSs after exercise; 32.2% before, during, and after exercise, and 13.3% before exercise. Significant differences were observed based on referee type (*p* = 0.014), with a higher prevalence of ingestion before, during, and after exercise among MRs than among ARs (OR = 1.63 [1.20–2.20]).

**Figure 1:**
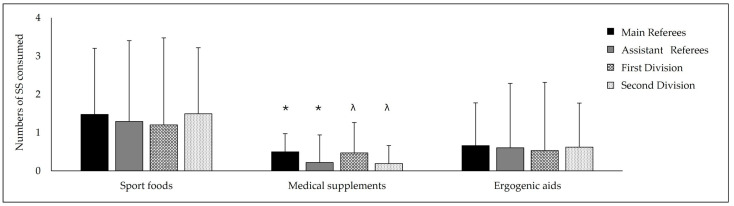
Numbers of Group A SSs as established by the AIS consumed by those in different subcategories. Data presented as M ± SD. *: Statistical differences between main and assistant referees; λ: statistical differences between First vs. Second Spanish Divisions; statistical differences fixed at *p* < 0.05.

Concerning the numbers of SSs consumed, the average intake was 3.37 ± 3.29, with no significant differences found between referee type (MRs: 3.63 ± 2.60 vs. ARs: 3.24 ± 3.63; *p* = 0.628) or category (First Spanish Division 3.63 ± 2.60 vs. Second Spanish Division: 3.12 ± 2.52; *p* = 0.661), or their interaction (*p* = 0.159). When classifying SSs using the AIS evidence categories (ABCD system), a significant difference was observed in the consumption of the medical supplement subgroup (Group A), with MRs being more likely than ARs (*p* = 0.016; OR = 3.02 [1.20–7.60]) in the First Spanish Division to consume them. MRs were more likely than those in the Second Spanish Division MR (*p* = 0.041; OR = 2.59 [1.02–6.55]) to consume these supplements. Moreover, a subanalysis of the medical supplements showed a higher average intake by MRs than by ARs (*p* = 0.023) and by those in the First Spanish Division compared to those in the Second Spanish Division (Figure 1).

**Figure 2:**
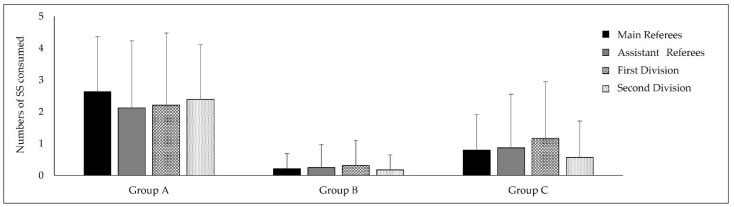
Numbers of SSs consumed by the different categories established by the AIS. Data are presented as M ± SD.

No statistical differences were found in the quantitative analysis of SS consumption across the different groups established by the AIS (i.e., Groups A, B, and C) (*p* > 0.05) (Figure 2).

Whey protein was the SS the most consumed by the sample (45.3%), followed by creatine (33.0%), sports bars and sports drinks (28.3%), and caffeine (19.8%). Significant differences were found in the consumption of glutamine, with a higher prevalence among MRs compared to ARs (*p* = 0.042; OR = 3.61 [0.98–13.27]) (Table 2).

## 4. Discussion

The aim of this study was to determine the consumption of SSs by establishing differences between competition levels (First Spanish Division vs. Second Spanish Division) and referee type (i.e., main referees vs. assistant referees) during the 2021–2022 football season. Previously, Malaguti et al. [17], in a study mixing professional with amateur football referees and assistant referees, found that referees reported insufficient knowledge of dietary supplements (not reporting the dietary supplements most consumed or the differences between main referees and assistant referees) and found a higher frequency supplements’ consumption without the supervision of a nutritionist or a medical doctor. However, the current study is novel because it reflects the prevalence of the use of supplements specifically in the First and Second Spanish Divisions and between referee type (i.e., main referees vs. assistant referees). It is interesting to mention that main and assistant referees reported similar SS consumption (84.0%), in agreement with previous studies in male football players who exhibited a consumption rate of 87.2% [7].

Regarding the anthropometric and personal characteristics of the referees, a significant difference was found between the MRs and ARs regarding age. Thus, we found that referees in the First Spanish Division were older than referees belonging to the Second Spanish Division. This difference may be explained by referees of the First Spanish Division needing to previously officiate in the Second Spanish Division. The percentage of those who advance from the Second to the First Spanish Division is very low; on average, it takes several years to be promoted between categories. In addition, the MRs and ARs of the First Spanish Division showed statistical differences in height and weight compared with their Second Spanish Division counterparts. These findings show that the physical fitness of elite referees could impact the category in which they arbitrate, as previously hypothesized [3].

The consumption of SSs could offer various advantages to football referees, such as enhancing cognitive and physical performance during matches [24], improving recovery between training sessions [25], and reducing the risks of injury and illness [26]. In the current study, different reasons were reported by MRs and ARs for consuming SSs, such as increasing sport performance (49.6%) and health status (38.2%), which is in agreement with findings previous articles in team sports athletes [7,8,9], which did not find statistical differences between referee type or referee category in relationship to the aims of their ingestion. Regarding the location of purchase, 41.0% of the sample purchased SSs on the Internet, whereas 19.7% purchased them from a pharmacy, and 17.2% from specialized stores. The Internet has been frequently cited as a preferred source for purchasing sports supplements [27,28]. This trend suggests that many of the surveyed referees might have been at risk due to the lack of global regulation on dietary supplements. The risk of contamination, the insufficient information on proper use, and the lack of scientific basis increase the likelihood of improper or excessive supplement consumption or accidental doping, especially with the unregulated online market [29]. Several studies have highlighted the dangers of online purchases, as sports supplements may contain undisclosed substances, incorrect dosages, or other contaminants, risking athletes’ health, performance, and careers [30,31,32].

Related to the advisor, the most frequent was a dietitian/nutritionist (D-N) (31.7%) followed by a physical trainer (25.4%), and another referee (15.9%). These findings indicate that professional referees consult a D-N as the first option, different from amateurs or university athletes who consult nonspecialized figures in the nutrition field, such as strength and conditioning coaches [21,33], before consulting a professional D-N or medical doctor. It is crucial to emphasize proper sources of advice, as misinformation and poor guidance can result in the use of non-evidence-based supplements or, even worse, can lead to unintentional doping and health risks [30,31]. Moreover, athletes who rely on D-N as their primary source of nutritional information tend to have healthier eating habits, a better grasp of nutrient timing, and make more scientifically informed choices regarding SSs [30,34].

The outcomes of the AIS classification of SSs showed a higher consumption of Group A SSs, with seven of the top ten SSs used belonging to this group [14]. Moreover, significant differences were observed between MRs and ARs exclusively in the use of medical supplements from Group A. However, no significant differences were found in the other subcategories of Group A (sports foods and performance supplements) or in the supplements from Groups B and C. The higher use of medical supplements among the different types of referees (i.e., MRs vs. ARs) could be attributed to the greater demands in total distance covered, high-intensity running actions [5], as well as higher cardiovascular demands [4] for MRs. These increased physical demands may necessitate nutritional supplementation that can be obtained through SS intake. Regarding the prevalence of SS consumption among referees by category and competition, whey protein (45.3%), creatine (33.0%), and sports drinks/sport bars (28.3%) were the most used SSs. All of them are in Group A.

Whey protein was the most consumed SS, and it has been established that its ingestion has a net effect of increasing muscle mass in elite football players [35] and may enhance performance recovery following team sport activity, despite an attenuation in indirect markers of muscle damage [36], which could partially explain the high consumption in Spanish football referees. Creatine supplementation has been shown to increase intramuscular creatine concentrations, thereby favoring the phosphagen energy system [37], which could explain the observed improvements in high-intensity exercise performance with its consumption, potentially enhancing referees’ activity. Sports drinks and sport bars were the third most consumed SSs, which were likely popular due to their capacity to maintain adequate fluid balance during refereeing activities. Additionally, they provide carbohydrates that sustain carbohydrate oxidation and blood glucose levels while reducing the use of muscle and liver glycogen stores during football referring activity [38,39]. Additionally, it is worth mentioning that glutamine was the only sports supplement with a higher prevalence of consumption that showed statistical differences between MRs and ARs. Glutamine is one of the most abundant amino acids in the body and is synthesized by skeletal muscle [40]. Glutamine ingestion can provoke increases in sports performance, enhance recovery, and reduce muscle damage [41], which could partially explain the difference between main and assistant referees. However, it is worth mentioning that a meta-analysis by Ramezani Ahmadi et al. (2018) established that glutamine supplementation has no effect on athletic performance, immune system, or body composition [42]; thus, glutamine should be taken with caution by referees.

The football nutritional program should prioritize a ‘food first’ approach, using supplements solely to meet specific health and performance objectives. Supplement dosages and durations should be meticulously recorded, with their effects—both positive and adverse—closely monitored [43]. While most of the supplements consumed by the sample belonged to Group A, which are those supported by scientific evidence, referees are not utilizing other supplements with proven performance-enhancing potential, such as sport gels, β-alanine, or nitrate [11,13,40]. Additionally, although further research is needed, supplements such as vitamin D, creatine monohydrate, collagen, and omega-3 (fish oil) could be promising to include in nutritional strategies for the rehabilitation of musculoskeletal injuries in athletes [44].

The use and sale of SSs warrant significantly greater attention than they currently receive. Unfortunately, there is no government body regulating the safety or efficacy of these products, and there is no oversight regarding the validation of their purported functions [45]. The standardization and categorization of SSs are essential for effective regulation. The risks associated with SS usage, their effective application, and the optimization of nutrient intake from food to minimize or eliminate reliance on supplements represent critical areas for research and education [18]. Understanding the effects of sports supplements is particularly crucial due to their potential impact on athletes’ performance and health, including that of football referees. Consequently, it is imperative for professionals advising athletes to possess comprehensive knowledge of responsible supplement use. This knowledge should emphasize three key aspects, efficacy, safety, and legality, as these factors underpin most of the issues associated with SSs in sports [12].

Given the rapid evolution of sports nutrition, staying updated with the relevant literature can be challenging. In 2017 alone, 2082 articles were published with the keywords “sports nutrition” [31]. To address this, several associations and expert groups regularly review and consolidate the current scientific evidence on the efficacy of the existing supplements. Notable among these are the Australian Institute of Sport (AIS) [14], the International Olympic Committee (IOC) [12], and the International Society of Sports Nutrition (ISSN) [46].

Despite the widespread use of supplements among athletes, their application is often inadequate. Therefore, conducting cost–benefit analyses of their proper and responsible use, alongside nutritional assessments that consider the individual context and needs of athletes, is fundamental [45]. Additionally, the consumption of supplements must not be viewed as a substitute for poor dietary choices or inadequate nutrition [47]. Furthermore, the indiscriminate use of SSs raises significant concerns and necessitates educational interventions from an early age for athletes, coaches, and family members [31].

Although, to the best of our knowledge, this is the first study conducted on SS consumption among professional referees, the current investigation has several limitations that must be considered when evaluating our findings. Firstly, the sample size in this study was exclusively composed of male MRs and ARs; therefore, future studies should be carried out exclusively with women referees to establish the SS consumption of this population segment. Secondly, although this study was carried out with referees from one of the most important football leagues in the world (Spanish Football League), it is necessary to replicate these studies with referees from other countries around the world to be able to observe whether the patterns of SS consumption are repeated in other leagues. Thirdly, the results of this study are based solely on the SS consumption of elite Spanish referees during the 2021–2022 season. Therefore, it cannot be assured that the consumption of supplements would be replicated in subsequent years. Additionally, a future line of research could involve conducting a similar analysis based on the athletic performance and physical fitness tests of referees, as this would add value by identifying which supplements and usage patterns are associated with different performance levels.

## 5. Conclusions

According to our findings, almost 84.0% of the main referees and assistant referees who participated in this study consumed at least one SS. The most consumed supplements were whey protein, with a prevalence of 45.3%, followed by creatine (33.0%), sports bars and sports drinks (28.3%), and caffeine (19.8%). Regarding the differences between category (i.e., First Spanish Division versus Second Spanish Division) and referee type (i.e., main vs. assistant referees), only differences in medical supplements were found.

## Figures and Tables

**Table 1:** Characteristics of the sample based on the referee type and level of competition.

		Competition					
Variable	Referee Type	First Spanish Division (*n* = 49)	Second Spanish Division (*n* = 57)	Total	Referee Type	Competition	Referee Type· Competition
Age (years)	MR	37.65 ± 9.44	36.30 ± 4.49 *	36.88 ± 3.39 *	F = 4.997 *p* = 0.001	F = 11.235 *p* = 0.028	F = 4.344 *p* = 0.040
	AR	37.51 ± 5.26 ^λ^	31.65 ± 3.68 *^,λ^	34.50 ± 5.37 *			
	Total	37.57 ± 6.38 ^λ^	33.45 ± 4.58 ^λ^	35.35 ± 5.84			
Height (cm)	MR	180.50 ± 4.35	181.86 ± 4.21 *	181.29 ± 4.27 *	F = 9.448 *p* = 0.003	F = 0.449 *p* = 0.482	F = 4.203 *p* = 0.043
	AR	179.45 ± 5.26 ^λ^	176.66 ± 5.35 *^,λ^	178.01 ± 5.46 *			
	Total	179.79 ± 4.96	178.67 ± 5.53	179.19 ± 5.28			
Weight (kg)	MR	75.31 ± 6.87 *	74.98 ± 4.18 *	75.11 ± 5.39 *	F = 8.808 *p* = 0.004	F = 0.403 *p* = 0.836	F = 0.004 *p* = 0.949
	AR	71.56 ± 6.39 *	71.39 ± 6.30 *	71.48 ± 6.30 *			
	Total	72.79 ± 6.22	72.77 ± 5.81	71.48 ± 6.30			
Body mass index (kg/m^2^)	MR	23.1 ± 2.32	22.7 ± 1.10	22.9 ± 1.71	F = 1.573 *p* = 0.213	F = 0.082 *p* = 0.775	F = 3.265 *p* = 0.074
	AR	22.2 ± 1.46	22.8 ± 1.28	22.5 ± 1.40			
	Total	22.5 ± 1.82	22.8 ± 1.21	22.7 ± 1.52			
Weekly	MR	6.13 ± 1.86	5.86 ± 1.13	5.97 ± 1.46	F = 0.039 *p* = 0.844	F = 0.605 *p* = 0.438	F = 0.045 *p* = 0.833
training	AR	6.12 ± 1.06	5.97 ± 1.29	6.04 ± 1.18			
sessions	Total	6.12 ± 1.35	5.93 ± 1.22	6.02 ± 1.28			

Data are expressed as M ± SD. *: Statistical differences between main referees (MRs) and assistant referees (ARs) of the same level of competition; ^λ^: statistical differences between First and Second Spanish Divisions for the same referee type; statistical differences fixed at *p* < 0.05.

**Table 2:** SSs with a higher prevalence of consumption according to the referee type and competition level.

Sport Supplement AIS Category		Sport Supplement	Total	Referee Type				Referee Category			
				MRs (*n* = 38)	ARs (*n* = 68)	*p*-Value	OR	First Spanish Division (*n* = 49)	Second Spanish Division (*n* = 57)	*p*-Value	OR
**Group A**	Sport foods	Sport bars	28.3%	36.8%	23.5%	0.179	1.90 [0.80–4.50]	22.4%	33.3%	0.280	0.58 [0.24–1.38]
		Sport drinks	28.3%	36.8%	23.5%	0.179	1.90 [0.80–4.50]	20.4%	35.1%	0.130	0.47 [0.20–1.15]
		Sport gels	13.2%	5.3%	17.6%	0.071	0.26 [0.06–1.23]	16.3%	10.5%	0.379	1.66 [0.53–5.16]
		Whey protein	45.3%	42.1%	47.1%	0.623	0.82 [0.37–1.83]	42.9%	47.4%	0.642	0.83 [0.39–1.80]
	Medical sup.	Multivitamins	12.3%	18.4%	8.8%	0.149	2.33 [0.72–7.54]	16.3%	8.8%	0.237	2.03 [0.62–6.67]
	Ergogenic aids	Creatine	33.0%	34.2%	32.4%	1.000	1.09 [0.47–2.52]	30.6%	35.1%	0.625	0.62 [0.36–1.85]
		Caffeine	19.8%	23.7%	17.6%	0.455	1.45 [0.55–3.83]	12.2%	26.3%	0.070	0.391 [0.14–1.10]
**Group B**		Vitamin C	11.3%	13.2%	10.3%	0.655	1.32 [0.39–4.49]	14.3%	8.8%	0.372	1.73 [0.51–5.86]
**Group C**		Magnesium	14.2%	15.8%	13.2%	0.717	1.23 [0.40–3.76]	16.3%	13.3%	0.551	1.39 [0.47–4.17]
		Glutamine	10.4%	18.4%	5.9%	0.042 *	3.61 [0.98–13.27]	12.2%	8.8%	0.559	1.45 [0.41–5.08]

*: Statistical differences between groups; Statistical differences fixed up *p* < 0.05.

## Data Availability

Data are available at the research data repository of the University of Seville.
